# What Must I Do to Get Well?
*"What must I do to get well, and how can I keep so?" By one who has done it. An exposition of the Salisbury treatment. Sampson Low, Marston, Searle, and Rivington, Limited, St. Dunstan's House, Fetterlane, Fleet-street, E.C. 1889.


**Published:** 1889-04-20

**Authors:** 


### What Must I Do to Get Well?*

The title-page of this book is without a name—"by one who has done it"—but the preface is signed E. Stuart, so it may be presumed that E. Stuart is the "one who has done it." The book professes to be an exposition of the Salisbury treatment. This treatment is based on the proposition that improper alimentation is the cause of disease, excepting when disease originates from accident, poisons, or infection. And the treatment is thus explained. "If you desire to get well you will take four pints of hot water a day, and restrict for a time your diet to minced beef only." The best times for the regular meals are 8 a.m., 1 p.m., and 6 p.m., and the best time for taking hot water are about 6.30 a.m., 11.30 a.m., 4.30 p.m., and 9 p.m. The water must not be swallowed quickly, but sipped slowly. The object and use of the hot water is stated as follows: It washes out the stomach; it stimulates the liver to activity, and prevents the bile from going "fooling around"; it causes a flow of urine; it liquifies and purifies the blood; it lightens the work of the heart; it washes out uric acid in the joints; it diminishes pain, and fulfils other beneficent offices." In short, it does everything which every patent medicine has been asserted to do from Holloway's pills downwards. After reading what hot water does, a diet of beef would scarcely seem necessary, but those who go in for the Salisbury treatment must also eat nothing but "lean beef, chiefly minced," which "should be cut from the round or flank above the hock." Now human nature is but weak, and we can readily imagine the patient beginning to rebel against such a diet, and to think better the disease than such a cure. The author admits that "it is a hard diet," and fully exercises all one's force of will, but he exhorts to perseverance, and "you will get to like it." The author now sees no doctors, and would apparently scorn the stock prescription of the fashionable physician: "ginger to warm, salvolatile to hold up, and soda to correct."

Well, we quite agree with the author that plenty of fluid is required in the system. Uric acid requires about 800 times its bulk of water to hold it in solution, and if it is not dissolved, it may be the cause of gout, gravel, and other less recognisable troubles. But, on the other hand, the amount of uric acid formed in the system differs in various people, and certainly all do not require the same proportion of fluid. Again, there is really no reason why the necessary amount of fluid should not be introduced into the system in a more palatable form than hot water; weak tea, for instance, or milk and water, or even weak beer, or well-made toast water. But if anyone prefers to drink a pint of hot water at 6 a.m., at 11.30 a.m., at 4.30 p.m., and at 9 p.m. we have no objection to offer. It will probably not do him any harm (although rather irksome), and it may do good. But when we are told to live on minced beef we become sceptical, and seek for some physiological or other reason why we should do so. Among the many sayings rightly or wrongly attributed to Sydney Smith, is "a man who eats a plain joint is only one remove from a cannibal," and now we are told to live on plain beef altogether. By structure man is both herbivorous and carnivorous, or, expressed otherwise, omnivorous. Therefore, what would be healthy alimentation for purely herbivorous and purely carnivorous animals would be unhealthy alimentation for man, since he partakes structurally of both the herbivora and carnivora, and belongs to the omnivora. Now we join issue thus far with the author, that there are times and diseases when a meat diet is desirable or even necessary; in some maladies of children, for instance, when milk is not well digested. But when we are told to keep our health by a meat diet, we recollect the truthful maxim *in medio tutissimus ibis*, and would certainly recommend a mixed diet. The author condemns vegetarianism as "a harmful delusion," and so it would be if anyone lived altogether on vegetables; but this few, if any, achieve; for eggs, milk, and butter are taken by vegetarians, even by the Hindus, thus converting so called vegetarianism into a mixed diet.

We cannot avoid thinking that the Salisbury hot water and minced beef treatment owes much of the success which it may have attained to the accompanying *regimen*. You must drop all stimulants; you must not drink with food, which is a pernicious habit; you must not swallow hard lumps; you must live in the climate that suits you best; you must have your teeth in good order, and, above all, you must have *faith*, and not use "bad language" if you don't get well quickly. Among the diseases for which the Salisbury treatment is said to be beneficial are Bright's disease, diabetes, obesity, rheumatism, tumours, neuralgia, pleurisy, dropsy, and cancers, which are said "to be curable!" After this we close the book with the reflection that, as Ophelia says of her rue, there are two kinds of people who take their good or evil fortune with a difference. No truer saying was ever invented than "What is one man's meat is another man's poison." "To quack of universal cures" has ever been the privilege of the Briton. It was Southey who said that man was a dupeable animal. As regards medicine and disease, we fear posterity will think us quite as foolish as we think our ancestors. The sooner the public can be brought to believe that there is no universal cure, that every person is by temperament and idiosyncracy differently constituted, and that therefore he or she requires a different treatment and diet—the sooner the public believe this the better it will be for the general health, and the worse for universal cure providers. While admitting that the Salisbury treatment may, either practised partially or wholly, sometimes prove beneficial, we must object to a general application of this or any other system.

* "What must I do to get well, and how can I keep so?" By one who has done it. An exposition of the Salisbury treatment. Sampson Low, Marston, Searle, and Rivington, Limited, St. Dunstan's House, Fetter-lane, Fleet-street, E.C. 1889.

